# Mantle Zone Lymphoma With Prostate Gland Enlargement: A Case Report

**DOI:** 10.7759/cureus.32045

**Published:** 2022-11-30

**Authors:** Krishna Doshi, Anjana Chandran, Yanxia Li, Jacob Bitran

**Affiliations:** 1 Internal Medicine, Advocate Lutheran General Hospital, Park Ridge, USA; 2 Hematology and Oncology, Advocate Christ Medical Center, Oak Lawn, USA; 3 Pathology, Advocate Christ Medical Center, Oak Lawn, USA; 4 Hematology and Oncology, Advocate Lutheran General Hospital, Park Ridge, USA

**Keywords:** non-hodgkin's lymphoma, oncology, genitourinary cancer, prostate enlargement, mantle zone lymphoma

## Abstract

The prostate is a rare site for the occurrence of lymphoma and accounts for 0.1% of newly diagnosed lymphomas. Patients with prostatic lymphoma exhibit similar symptoms to that of benign prostatic hyperplasia and prostate carcinoma, such as obstructive and irritative urinary signs. Therefore, due to its rare occurrence, it is difficult to distinguish between primary lymphoma of the prostate and other etiologies. We present a unique case of an elderly individual who presented with enlarged prostate and was found to, concurrently, have mantle zone lymphoma of the prostate.

## Introduction

Mantle cell lymphoma (MCL) comprises an uncommon subtype of non-Hodgkin's lymphomas (NHLs). It results from the malignant transformation of a B lymphocyte in the mantle zone of the lymph node follicle. Most of the patients who have MCL have a genetic predisposition on chromosomes 11 and 14 called reciprocal translocation. The exchange of genetic material between the two chromosomes occurs at the cyclin D1 gene site on chromosome 11. This gene controls the antibody formation on chromosome 14. Therefore, upon exchange of this material, there is increased production of cyclin D1 which results in increased cell growth and production. The diagnosis of MCL is made upon tissue examination of the lymphoid cells which demonstrates cluster of differentiation 20 (CD20) marker on B cell, presence of translocation between chromosome 11 and chromosome 14, and overexpression of cyclin D1 protein in cells. Once the extent of the disease is determined, a Mantle Cell International Prognostic Index (MIPI) score is used to develop a treatment plan for the patient. The score is calculated using factors present at the time of diagnosis - age, performance status (ability to perform activities of daily life), lactate dehydrogenase levels, and leukocyte count. Additionally, the presence of several tumor markers can help determine the prognosis for the patient. Which includes markers of cell proliferation (Ki-67), gene expression profiling; minimal residual disease (MRD); MCL cell type; peripheral blood monocyte count (AMC) at the time of diagnosis, and beta-2 microglobulin level. MCL is generally considered an aggressive subtype of NHL. Treatment usually involves rituximab in combination with other medications. Several standard practices continue to use rituximab, cyclophosphamide, doxorubicin hydrochloride, Vincristine sulfate, and prednisone as a steroid (R-CHOP) as their primary treatment plan. It works to reduce inflammation and destroy target tumor DNA. Other treatment regimens include Rituxan in combination with cyclophosphamide, vincristine, doxorubicin, dexamethasone, and high-dose methotrexate with leucovorin rescue (R-HyperCVAD). More recently, FDA approved the VcR-CAP treatment which includes - bortezomib (Velcade), Rituxan, cyclophosphamide, doxorubicin (Adriamycin®), and prednisone for previously untreated patients with MCL. For older fit patients who have comorbidities, bendamustine and Rituxan (BR) is an alternative. Primary NHLs of the prostate account for 0.09% of prostatic neoplasia and 0.1% of all NHLs [1]. MCL of the prostate is even rarer [1]. There are only five documented cases in literature of this. We report a unique case of MCL with prostate enlargement with classic features [2,3].

## Case presentation

**Figure 1:**
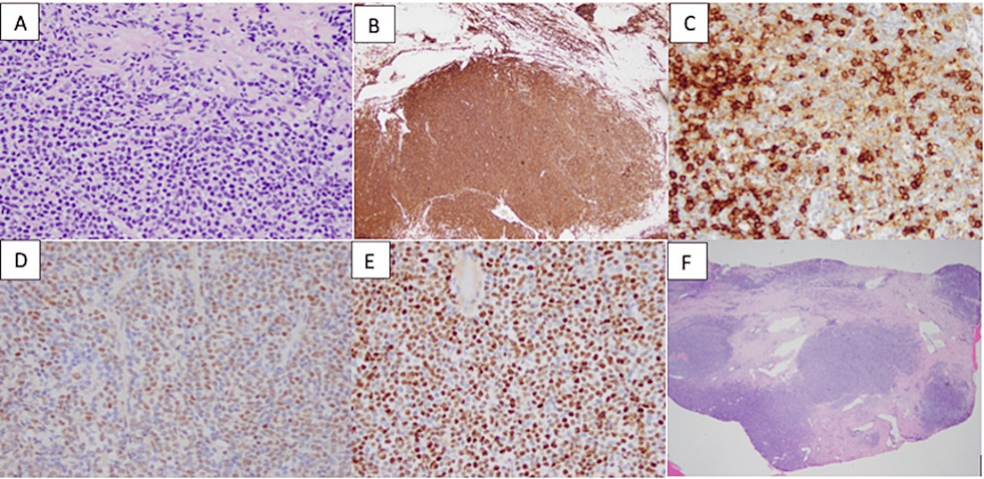
Pathology slides from the prostate biopsy. The images show (A) the infiltrating lymphocytes are intermediately sized with abundant cytoplasm and irregular nuclear contour (H&E, x200); (B) dense lymphocytic infiltrate in the prostate parenchyma. No residual prostatic glands are identified (H&E, x20); (C) B cells are positive for SOX 11; (D) the B cells are positive for cyclin D1; (E) CD5 is strongly positive in the T cells and weakly positive in the B cells (IHC, x400); and (F) the infiltrating lymphocytes are B cells, positive for CD20 (IHC, x50). IHC: immunohistochemistry

**Figure 2:**
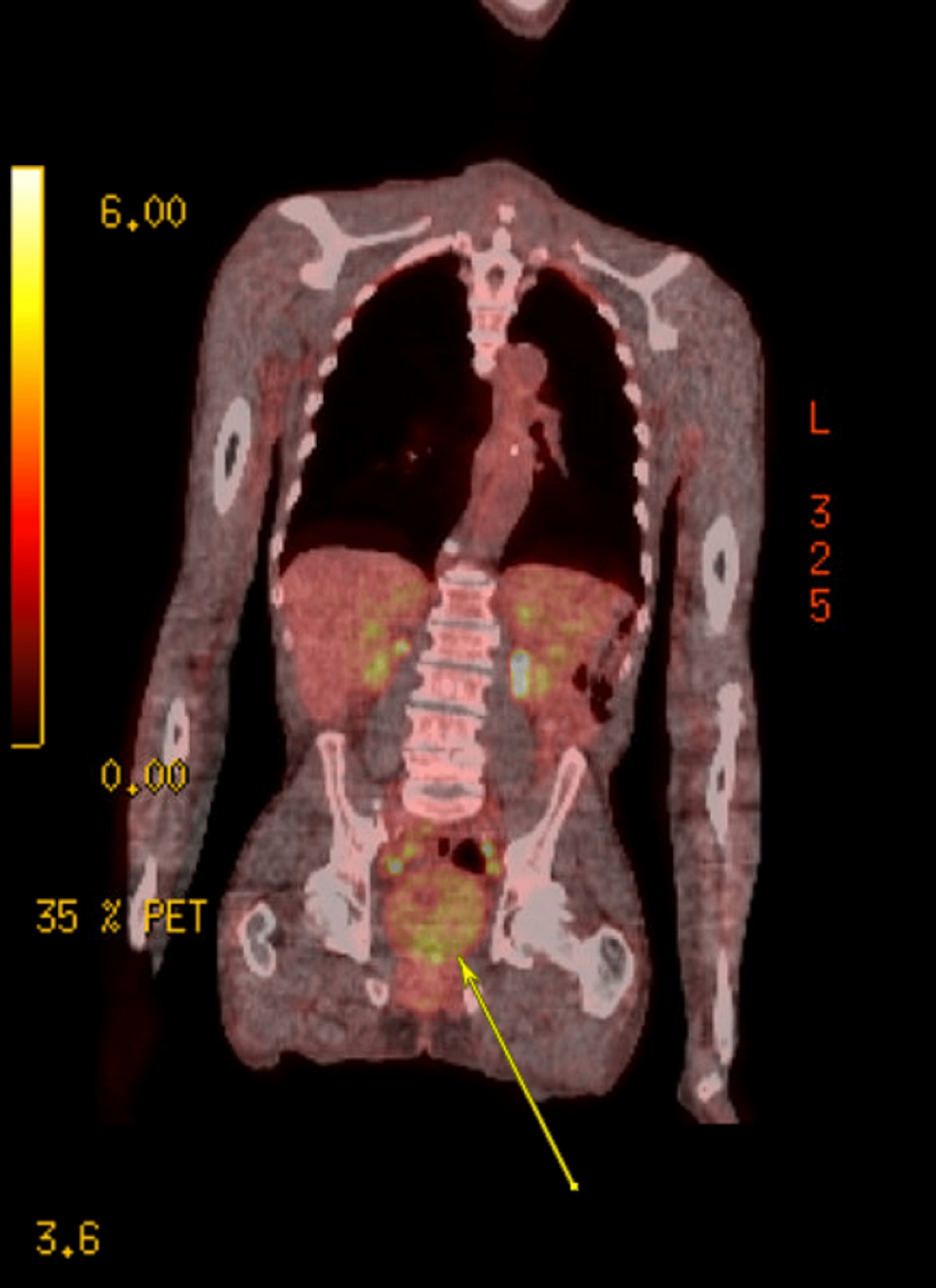
PET-CT scan demonstrating the extent of disease within the prostate.

An 81-year-old male with past medical history significant for benign prostatic hyperplasia, bilateral hydronephrosis, congestive heart failure, essential hypertension, and chronic obstructive pulmonary disease presented to the emergency department in May 2022 for the chief concern of urinary retention and hematuria. The patient reported having several episodes of acute urinary retention in the past requiring a Foley catheter. The results of the clinical examination were normal. Urine analysis and urine culture were negative. White blood cell count was elevated to 13,300 per microliter, and creatinine and blood urea nitrogen (BUN) were within normal range. Prostate-specific antigen (PSA) was elevated to 8.8 nanograms per milliliter. Lactate dehydrogenase was normal. Ultrasound of the kidney and bladder showed bilateral hydronephrosis and prostate measuring 7.4x6.7x6.2 cm. The patient was discharged with Foley catheter with the plan to follow-up with urology as an outpatient. Subsequently, transurethral resection of the prostate was performed which showed mantle cell lymphoma, classic type. The prostate chips were CD20+ cells with co-expression of cyclin D1, SOX11, and focal weak CD5. CD3 and CD5 also highlight scattered T cells (Figures 1A-1F). Subsequently, the patient was seen by hematology for further evaluation and workup. The physical findings were remarkable for the absence of any peripheral adenopathy. Positron emission tomography (PET) scan was ordered which showed hypermetabolic bilateral cervical, axillary, and external iliac lymphadenopathy and a large multifocal hypermetabolic prostate mass with an index focal abnormal fluorodeoxyglucose (FDG) uptake towards the left anterior superior aspect of the prostate gland (Figure 2). The patient was started on bendamustine-rituximab. Following his first cycle, all his urinary symptoms disappeared.

## Discussion

Mantle zone lymphoma (MCL) of the prostate is rare with only five reported cases in the current literature [2,3]. MCL incidence is five cases per 100,000 individuals per year and more likely in males than females [4]. It usually presents at the median age of 60 years old and the median survival in these patients is three to five years [4]. The cytogenic abnormality underlying MCL involves t(11;14) (q13;32) translocation between immunoglobulin heavy chain IGH gene on chromosome 14 and the BCL1 locus on chromosome 11 [5]. Which results in the dysregulation of the expression CCND1 and increased half-life of cyclin D1 [5]. This, in turn, leads to loss of cycle suppressive effects of RB1 protein and p27Kip1 leading to the development of MCL [6]. In MCLs, deregulation of BCLl/cyclin D1 may cause the G1 cell cycle checkpoint to be disrupted, leading to continued proliferation [7,8]. In terms of clinical characteristics, morphologically defined MCLs have been shown to resemble low-grade stage III or IV NHL at disease presentation, with a low frequency of complete remission and a median survival of approximately five years [9].

Previous literature has challenged the existence of primary prostatic lymphoma due to the paucity of lymphoid tissue in the prostate [10]. However, due to the existence of rudimentary lymphoid nodules and extramedullary hematopoiesis in the prostate, prior histopathological cases have confirmed the existence of primary prostatic lymphoma [11]. Primary prostatic lymphoma has been defined by the criteria of Bostwick and Mann, which includes (1) symptoms attributable to prostatic enlargement, (2) prostate as the predominant site of involvement, and (3) absence of involvement of the liver, spleen, or lymph nodes within one month of diagnosis [10]. The retrospective review by Bostwick et al. demonstrated that primary lymphoma is rarer than secondary lymphoma of the prostate (35% vs 65%) [11]. This case is therefore unique to due its presentation of MCL with primary prostate involvement.

Diagnosing MCL with primary involvement of the prostate is challenging as it can be difficult to distinguish it from other lower genitourinary tract obstruction diseases. The usual systemic symptoms of fever, weight loss, and night sweats are uncommon in patients who are diagnosed with primary prostatic lymphomas. These symptoms usually manifest after disease dissemination [12]. The patient in this case did not demonstrate the classic symptoms, and kidney function in his labs was normal. Therefore, clinical suspicion of this diagnosis was low.

Since the occurrence of prostatic lymphoma is quite rare, the treatment options have not been optimized yet. Several treatment modalities have been reported in literature, including radical prostatectomy, chemotherapy, radiotherapy, or concurrent chemotherapy. Currently, treatment is performed using the standard cyclophosphamide, doxorubicin, vincristine, and prednisone (CHOP) regimen. There are sporadic reported cases of positive response to rituximab being employed for treatment [12]. Given the patient's age and co-morbidities, a different regimen was employed for treatment.

However, the study by Bostwick et al. of 62 patients showed that there is no significant difference in survival between patients receiving different types of therapies [11]. Furthermore, lymphoma-specific survival did not differ between primary and secondary involvement in contrast to patterns noted in bladder and ovarian cancers [11]. The prognosis of patients remains poor regardless of patient age, histological type, treatment, or clinical stage of disease at presentation [11].

## Conclusions

In conclusion, MCL of the prostate presents as prostatism which is rare. Therefore, diagnosing it at the initial presentation can be challenging due to the presence of similar symptoms in other genitourinary obstructive disorders. We described a unique case of MCL with primary prostate involvement and response to appropriate intervention. Hence, It should be on the differential when evaluating patients with chronic urinary problems to direct appropriate timely intervention.
